# User Experience of Generating PSPDPs on Portfolio Online Amongst Psychiatry Trainees and Trainers

**DOI:** 10.1192/bjo.2024.298

**Published:** 2024-08-01

**Authors:** Aradhana Gupta, Pallavi Chandra

**Affiliations:** 1 Black Country Healthcare NHS Foundation Trust, Walsall, United Kingdom; 2 Black Country Healthcare NHS Foundation Trust, Sandwell, United Kingdom

## Abstract

**Aims:**

The Royal College of Psychiatrists (RCPsych) introduced the new psychiatry training curriculum in February 2022. Since then there have been various updates in both the e-portfolio platform and curriculum requirements.

A survey was undertaken to understand issues experienced in navigating these changes by psychiatry trainees and supervisors within the Black Country Healthcare NHS Foundation Trust (BCHFT), specifically assessing the generation of Placement Specific Personal Development Plans (PSPDPs) for each training placement.

The aim of this study was to survey user experience and reflect on the results to identify how best to support trainees and supervisors in using PSPDPs, a key curriculum requirement, with greater ease and confidence.

**Methods:**

The survey comprised tailored questionnaires distributed to two cohorts- trainees (30) and supervisors (37) within the BCHFT. Anonymised responses were collected over one month. Likert scales were used to determine (a) confidence levels in setting up PSPDPs, (b) confidence in mapping activities to both PSPDPs and the curriculum, and (c) user-friendliness of RCPsych guidelines on this topic. Checklists and free-text responses were used to assess which support resources were being utilised by both groups. Suggestions were requested on how the whole process could be improved.

**Results:**

Amongst trainees (response rate 63%), 78% did not feel confident in setting up PSPDPs. 94.7% sought additional support in PSPDP setup, of which peer support was the most utilised (77.8%). Other resources included the RCPsych website and emails as well as supervisors. 58% of trainees lacked confidence in linking activities to PSPDPs and the curriculum. Only 10.5% of the trainees found the RCPsych Implementation Hub user friendly.

In the supervisor cohort (40% response rate), 64% of the trainers felt confident in guiding their trainees in setting up PSPDPs. 85% utilised support from various sources including the Implementation Hub (91.7%), trainees (58.3%) and peers (50%). 64.2% of supervisors found the RCPsych website user friendly.

**Conclusion:**

Common themes that emerged were that both trainees and supervisors felt the process of setting up PSPDPs was quite complex, with a confusing web interface. Resources on the RCPsych website required better signposting. Both cohorts felt they would like additional training e.g. step by step videos and training sessions (local peer trainee and supervisor run sessions were found useful).

This feedback has identified the importance of arranging local training sessions to improve engagement. Additionally, we hope that relaying this feedback to RCPsych may influence future systemic changes.

